# Clutter Elimination and Random-Noise Denoising of GPR Signals Using an SVD Method Based on the Hankel Matrix in the Local Frequency Domain

**DOI:** 10.3390/s18103422

**Published:** 2018-10-12

**Authors:** Wenda Bi, Yonghui Zhao, Cong An, Shufan Hu

**Affiliations:** School of Ocean and Earth Science, Tongji University, Shanghai 200092, China; bwd1996@tongji.edu.cn (W.B.); ancong@tongji.edu.cn (C.A.); 14_husf@tongji.edu.cn (S.H.)

**Keywords:** ground-penetrating radar, clutter, random noise, singular value decomposition, Hankel matrix

## Abstract

Ground-penetrating radar (GPR) is a kind of high-frequency electromagnetic detection technology. It is mainly used to locate targets and interfaces in underground structures. In addition to the effective signals reflected from the subsurface objects or interfaces, the GPR signals in field work also include noise and different clutters, such as antenna-coupled waves, ground clutters, and radio-frequency interference, which have similar wavelet spectral characteristics with the target signals. Clutter and noise seriously interfere with the target’s response signal. The singular value decomposition (SVD) filtering method can select appropriate singular values and characteristic components corresponding to the effective signals for signal reconstruction to filter the GPR data. However, the conventional time-domain SVD method introduces fake signals when eliminating direct waves, and does not have good suppression of random noise around non-horizontal phase axes. Here, an SVD method based on the Hankel matrix in the local frequency domain of GPR data is proposed. Different numerical models and real field GPR data were handled using the proposed method. Based on the power of fake signals introduced via different processes, qualitative and quantitative analyses were carried out. The comparison shows that the newly proposed method could improve efforts to suppress random noise around non-horizontal phase reflection events and weaken the horizontal fake signals introduced by eliminating clutter such as ground waves.

## 1. Introduction

Ground-penetrating radar (GPR) is a geophysical method using high-frequency electromagnetic waves to detect and locate targets and interfaces in underground structures [1]. With the advantages of simple operation, rapidity, economy, non-destructive detection, and high precision, GPR became one of the most important tools for shallow geophysical exploration, and it is widely used in civil engineering [2,3], environmental engineering [4], archaeological exploration [5], military exploration [6], and other fields. In the process of data collection from ground-penetrating radar, the receiver receives reflected signals from underground targets and interfaces, which belong to the effective target signals; at the same time, it is inevitable to receive clutter from various non-targets and random noise. Clutter is produced from direct coupling between the transmitter and receiver, fluctuation of the ground surface, reflected signals of the underground non-target objects, reflected signals of the interface between the air and ground surface, reflected signals of objects on the ground (such as cables, buildings, etc.) near the detection area, multiple waves, and so on. Due to the wide frequency range received by GPR antennas and the complicated collection environment, GPR signals are usually mixed with random noise, whose correlation with the adjacent traces is nearly zero [7].

The clutter and the target signals appear in the same time window and have the same spectral characteristics as the target signals, interlacing each other. The strong direct wave and the ground reflection signal mask the effective reflection signals of shallow targets, and reflected signals of objects on the ground and their multiple waves may also cover some effective reflection signals, affecting the detection of targets and interfaces. Random noise is generally poorly correlated, and its energy is generally weak when there is no strong electromagnetic interference from communication equipment, as the background distributed in the GPR data. The effective reflection signal is interfered with if the energy of the target reflection signal is weaker than that of the random noise. In general, the clutter and random noise in GPR data seriously affect the recognition of effective reflection signals of targets and interfaces; thus, it is necessary to adopt filter methods to eliminate these interferences after obtaining the measured GPR data. Furthermore, filtering methods cannot affect the response of the targets and must reduce the influence on effective signals as little as possible to ensure the effectiveness and accuracy of exploration using GPR.

Singular value decomposition (SVD) provides a convenient way of decomposing a matrix, which perhaps contains some data we are interested in, into the multiplication of three simple matrices, and with further decomposition, these three multiplied simple matrices can be written as the addition of various components with different degrees of linear correlation weighted by singular values [8]. GPR signals can be classified into three categories from the aspect of correlation: the first category involves the direct wave, reflected signals of horizontal interface, and their multiple waves with strong linear correlation; the second involves the effective reflection signal with weak linear correlation needed to be preserved in the filtering process; the last is random noise with almost no correlation. Thus, we can use the SVD algorithm to eliminate clutter and random noise by selecting appropriate singular values, and there were some successful applications. Fawzy Abujarad studied the SVD technique for clutter reduction and nonmetallic landmine detection of stepped-frequency GPR (SF-GPR) signals and achieved a good result for the automatic selection of SVD components [6]. Fangyuan Nan used the SVD technique and presented an approach for model-order determination and successfully reconstructed the total-time responses. The model order was quantitatively selected using spectral analysis of left singular vectors of the data matrix and of the emitted waveform [9]. Muhammad Mohsin Riaz proposed the SVD and fuzzy c-means (FCM)-based method to reduce clutter for ground-penetrating radar imaging. The scheme could discriminate clutter, target, and noise subspaces. They used fuzzy c-means to separate the overlapping boundaries of clutter, target signals, and noise, and obtained the target image using the weighted sum of different spectral components [10]. Tomasz M. Grzegorczyk proposed a two-pass GPR approach to detect recently buried nonmetallic scatters, whose signals were otherwise undistinguishable from surrounding clutter, using an algorithm based on a modified singular value decomposition approach, which was shown to perform significantly better than a direct signal cancellation approach [11]. Song presented a method using SVD to eliminate random noise and direct waves from GPR signals, and found that choosing appropriate singular values after SVD of the GPR data could validly and efficiently eliminate the random noise and direct wave, thereby improving the resolution of the GPR profile [12]. However, this method introduced horizontal fake signals when eliminating the direct wave, which covered the effective signal, perhaps due to the flat top of the curved phase axis. Chen Gaoxiang used SVD based on the careful analysis of the characteristics of ground-penetrating radar data to accomplish a method of automatically determining the number of singular values to eliminate coherent noise, thereby avoiding the trouble of judging the number of singular values for different data. The method was particularly suitable for the coherent noise elimination of GPR data obtained in the case of surface fluctuation [13]. However, for seismic data, the conventional time-domain SVD method does not have good suppression of random noise around non-horizontal phase axes, and an improved SVD algorithm based on the Hankel matrix could deal with this problem. Good results could be obtained because this algorithm can enhance the auto-correlation of the non-horizontal phase axis [14]. We can use this method to deal with the similar problem appearing in GPR data. Based on these research findings, this paper proposes an SVD filtering method based on the Hankel matrix in the local frequency domain of GPR data to eliminate clutter and random noise. We made a comparison between the new method and the conventional time-domain SVD method when handling simulated model data using qualitative analysis and quantitative analysis by calculating the power of the fake signal introduced by the processing. The GprMax2D V.2 software, which solves two-dimensional Maxwell equations based on the finite-difference time-domain (FDTD) technique, was used for numerical simulations [15]. Furthermore, we tested the newly proposed method on real field data obtained from an underwater archaeological investigation and an airport runway evaluation using GPR, and compared the results with the conventional time-domain SVD method and the mean-trace-removing method.

## 2. The SVD Filtering Method Based on the Hankel Matrix in the Local Frequency Domain

Let us assume the two-dimensional (2D) GPR data are denoted by *X*, and there are *m* traces in the GPR data and *n* sampling points in every trace (assuming that *n* > *m*). According to the theory of SVD, the matrix *X* can be written as

(1) X = USVT = [u1…un][σ10⋱σm  ⋮0 0][v1T⋮vmT],

where *U* and *V* are the left singular and right singular orthogonal matrices, respectively, where the size of *U* is *n* × *n* and the size of *V* is *m* × *m*; *S* is a diagonal matrix with a size of *n* × *m*; the elements σ_s_ (1 ≤ s ≤ *m*) of matrix *S* are the singular values of matrix *X*; and σ_1_ ≥ σ_2_ ≥ … ≥ σ_m_ > 0.

Equation (1) can be written as

(2) $$X\;=\;\sigma_{1}u_{1}v_{1}^{T}+\sigma_{2}u_{2}v_{2}^{T}+\cdots +\sigma_{m}u_{m}v_{m}^{T},$$

where, $u_{s}v_{s}^{T}$ denotes the *s*-th diagnostic component of matrix *X* with the same size as *X*. In general, several front singular values contribute largely to matrix *X* as the decreasing order of singular values. According to the characteristics of the GPR signal, the first few singular values correspond to components denoting the direct wave, reflected signals of the horizontal interface, and their multiple waves with strong linear correlation among the traces; and the smaller singular values correspond to components denoting the random noise with almost no correlation. Thus, matrix *X* can be written as

(3) $$X\;=\overset{k_{1}}{\underset{s=1}{\sum }}\sigma_{s}u_{s}v_{s}^{T}+\overset{k_{2}}{\underset{s=k_{1}+1}{\sum }}\sigma_{s}u_{s}v_{s}^{T}+\overset{m}{\underset{s=k_{2}+1}{\sum }}\sigma_{s}u_{s}v_{s}^{T}.$$

We can select the appropriate singular values (*k*_1_–*k*_2_) which denote the effective signals to reconstruct the GPR data matrix, before getting the GPR data without clutter and random noise. To eliminate clutters with strong linear correlation among the traces, we usually set *k*_1_ = 1, and, to suppress random noise, we usually delete the smaller singular values and preserve the preceding few larger singular values. It is important to note that, if there are horizontal interfaces underground, the reflected signals of these may also be eliminated when eliminating the direct wave. In this case, it may be an effective method to district the GPR profile and then use the SVD method.

The Hankel matrix is a matrix in which each ascending skew-diagonal from left to right is constant; thus, if the elements of the first column and last row are determined, the whole matrix can be determined. According to the characteristics of the Hankel matrix, we can arrange the GPR data in a slice of a Hankel matrix, and carry out the SVD algorithm for the Hankel matrix to denoise. This algorithm can enhance the auto-correlation of the non-horizontal phase axis and reduce the auto-correlation of the horizontal phase axis or the horizontal part on the non-horizontal phase axis (if it exists). The SVD algorithm can decompose the matrix into the addition of various components with different degrees of linear correlation weighted by singular values. Hence, the SVD method based on the Hankel matrix can improve the suppression of random noise around the non-horizontal phase axis and weaken the horizontal fake signals produced by eliminating the direct wave. The GPR data with *m* traces in each frequency slice of the frequency domain denoted by $a_{t}$ (1 ≤ *t* ≤ *m*) can be arranged in a Hankel matrix:

(4) $$H\;=\;\left[\begin{array}{cccc} a_{1} & a_{2} & \ldots & a_{m-p+1}\\ a_{2} & a_{3} & \ldots & a_{m-p+2}\\ \vdots & \vdots & \vdots & \vdots \\ a_{p} & a_{p+1} & \ldots & a_{m} \end{array}\right].$$

The processes of suppressing noise with the SVD method based on the Hankel matrix in the local frequency domain are listed below.

1. GPR data within a local window (window size = 3) sliding in the time domain are extracted and Fourier transformed.
2. A frequency slice in the bandwidth is extracted to form the Hankel matrix, whereby *p* = *m*/2 to ensure, as much as possible, that *H* is a square matrix.
3. SVD is applied to *H*, and appropriate singular values are selected to reconstruct the Hankel matrix. Attention needs to be paid to the complexity of the signal data in the frequency domain; thus, transposition of the right singular vector of the SVD in the frequency domain is conjugate.
  (5) $$H\;=\;\sigma_{1}u_{1}v_{1}^{H}+\sigma_{2}u_{2}v_{2}^{H}+\cdots +\sigma_{p}u_{p}v_{p}^{H}.$$
4. The Hankel matrix recovers the data in a frequency slice.
5. The same process is repeated for every slice in the frequency domain; then, the inverse Fourier transform is used to get the noise-suppressed data in the time domain.

## 3. Results

In this paper we tested three models using a numerical simulation to compare the conventional time-domain SVD method and the SVD method based on the Hankel matrix in the local frequency domain. Then, real field data obtained from an underwater archaeological investigation and an airport runway evaluation using GPR were also handled using the two SVD methods, as well as the mean-trace-removing method.

### 3.1. Numerical Simulation

#### 3.1.1. Horizontal Interface Model

**Figure 1:**
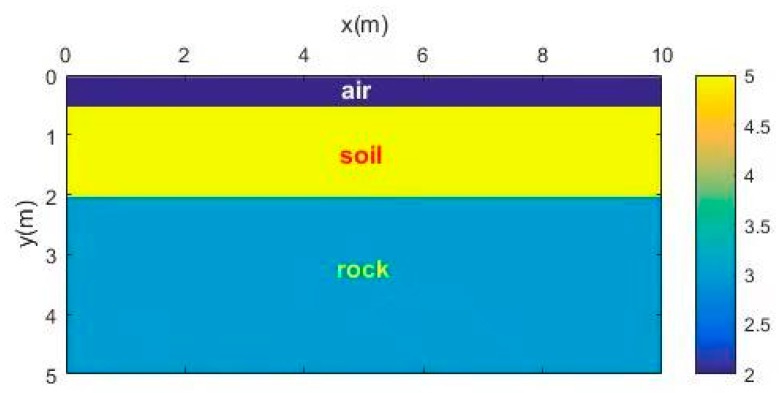
Horizontal interface model.

**Figure 2:**
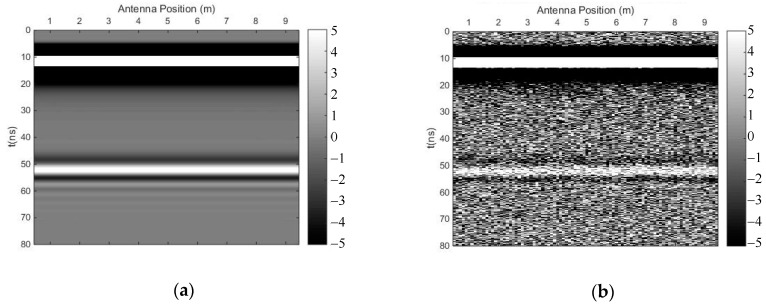
Ground-penetrating radar (GPR) profile of the horizontal interface model. (**a**) Original forward profile; (**b**) profile after adding Gaussian white noise; (signal-to-noise ratio (SNR) = 20).

Firstly, a simple model with a horizontal interface was used to test the noise-suppressing abilities of the time-domain SVD method and the SVD method based on the Hankel matrix in the local frequency domain. Figure 1 shows the simple 2D model with horizontal interfaces. There were two layers—the antennas of the GPR were placed in the air–soil interface, and the source function was a Ricker wavelet whose dominant frequency was 100 MHz. The original forward profile and the profile after adding Gaussian white noise (signal-to-noise ratio (SNR) = 20) are shown in Figure 2).

**Figure 3:**
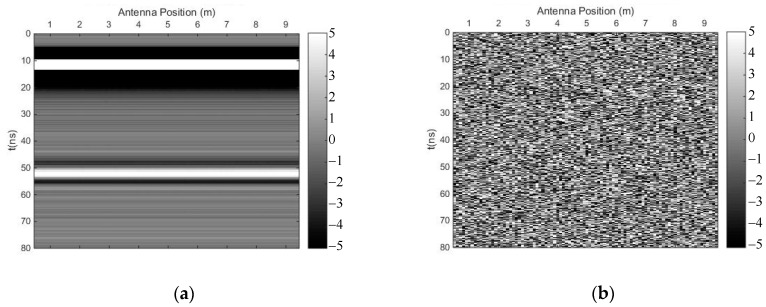
The results of the time-domain singular value decomposition (SVD) method. (**a**) Profile after using time-domain SVD; (**b**) eliminated profile.

**Figure 4:**
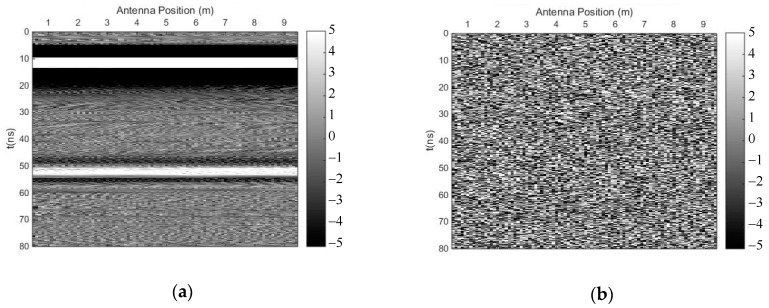
The results of the SVD method based on the Hankel matrix in the local frequency domain. (**a**) Profile after using the proposed method; (**b**) eliminated profile.

We carried out both methods to suppress random noise, selecting the first singular value to preserve the horizontal reflection events. Figure 3 shows the profile after using the time-domain SVD method, and the eliminated profile. Though this method could eliminate random noise effectively, it introduced many fake horizontal signals due to the weak linear correlation of some of the noise. Figure 4 shows the profile after using the SVD method based on the Hankel matrix in the local frequency domain, and the eliminated profile. This method did not perform as well as the time-domain SVD when eliminating random noise, but it did not introduce many fake horizontal signals, which may be regarded as the reflected signals of other horizontal interfaces. In the regions of 20–48 ns and 55–80 ns (*y*-axis), where many fake signals were introduced (Figure 3a), the power of the signals including the fake signals was calculated for comparison with the same regions in Figure 4a. The ratio was 0.32, indicating that the number of fake signals introduced by the time-domain SVD method were much lower than those introduced using the newly proposed method. The reason is that the Hankel matrix enhances the linear correlation of some noise; thus, when a signal with strong linear correlation is to be preserved, some noise will also be preserved.

#### 3.1.2. Undulating Interface Model

**Figure 5:**
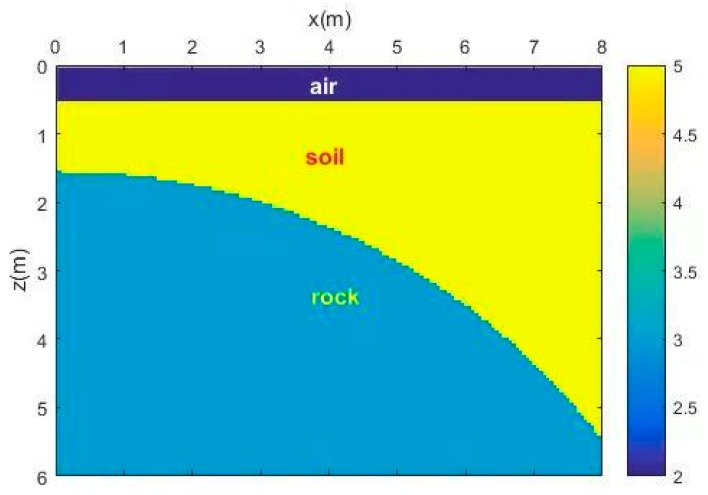
Undulating interface model.

**Figure 6:**
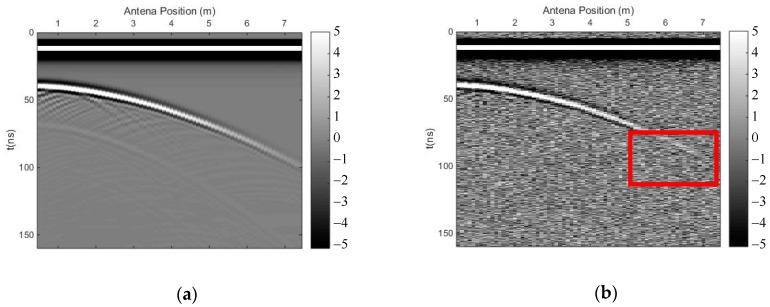
GPR profile of the undulating interface model. (**a**) Original forward profile; (**b**) profile after adding Gaussian white noise (SNR = 25).

Figure 5 shows the simple 2D model with an undulating interface of soil and rock. The antennas of the GPR were placed in the air layer, and the source function was a Ricker wavelet whose dominant frequency was 100 MHz. The original forward profile and the profile after adding Gaussian white noise (SNR = 25) are shown in Figure 6. The reflected phase axis of the interface is curved.

**Figure 7:**
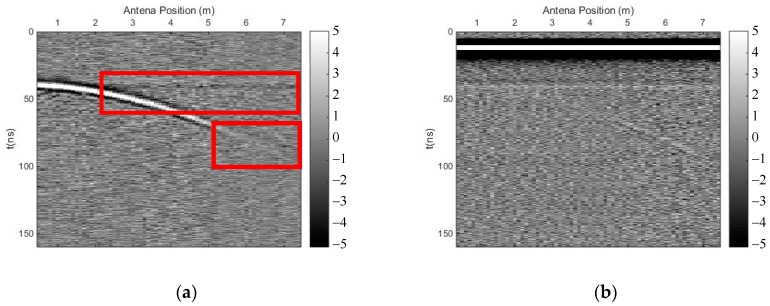
The results of the time-domain SVD method. (**a**) Profile after using time-domain SVD; (**b**) eliminated profile.

**Figure 8:**
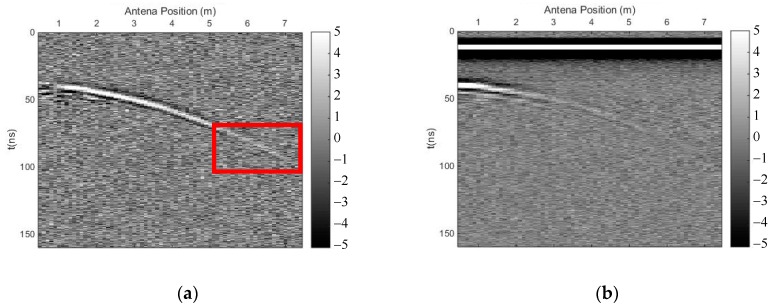
The results of the SVD method based on the Hankel matrix in the local frequency domain. (**a**) Profile after using the proposed method; (**b**) eliminated profile.

We carried out both methods to eliminate the direct wave and suppress random noise. Figure 7 shows the profile after using the time-domain SVD method, and the eliminated profile. The horizontal fake signal is shown in the red frame, perhaps due to the flat top of the curved phase axis, and the effective signal was also damaged, especially the deep effective signal. Figure 8 shows the profile after using the SVD method based on the Hankel matrix in the local frequency domain, and the eliminated profile. This method did not introduce a fake horizontal signal and the effective signal was also damaged, perhaps due to the varying gradient of the curved phase axis. In the regions where the fake signals were introduced in Figure 7a, the power of the signals including the fake signals was calculated for comparison with the same regions in Figure 8a. The ratio was 1.1, indicating that the fake signals introduced by the time-domain SVD method were much stronger than those introduced by the newly proposed method. The regions of 40–50 ns (*y*-axis) and 0–1 m (*x*-axis) of the profile were seriously damaged because the linear correlation of these regions was enhanced using the Hankel matrix; hence, when eliminating the clutter, these regions were damaged. Nevertheless, when using the new method to suppress noise, the deep effective signal with the curved phase axis was better preserved than when using the time-domain SVD method; thus, the deeper signal was distinguished more easily.

#### 3.1.3. Reinforced Concrete Model

**Figure 9:**
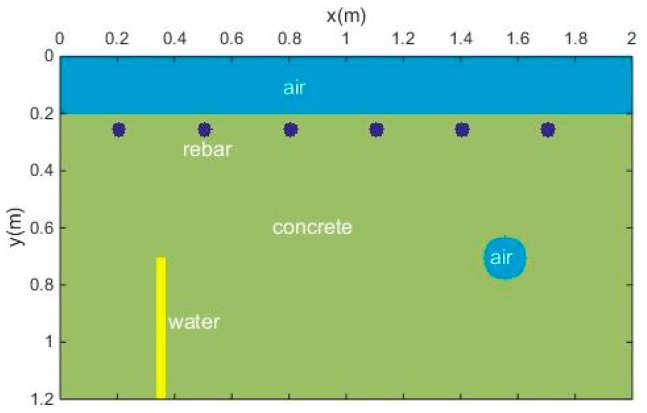
Reinforced concrete model.

**Figure 10:**
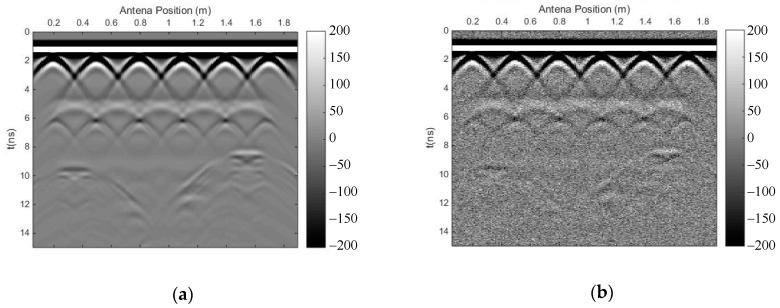
GPR profile of the reinforced concrete model. (**a**) Original forward profile; (**b**) profile after adding Gaussian white noise (SNR = 20).

Figure 9 shows a simulated 2D reinforced concrete model with some defects—a round pore and a fracture filled with water. The antennas of the GPR were placed in the air layer, and the source function was a Ricker wavelet whose dominant frequency was 900 MHz. The original forward profile and the profile after adding Gaussian white noise (SNR = 20) are shown in Figure 10. It can be seen that the signals of the rebar were covered by the strong direct wave and the reflected signal of the surface; thus, we could not calculate the depth of the rebar because we could not learn the travel time of the reflected signals of the rebar. The reflected signals of the air-filled hole and the water-filled fracture can be seen in the profile.

**Figure 11:**
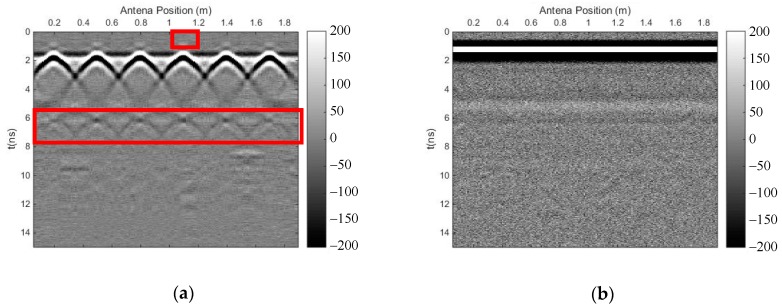
The results of the time-domain SVD method. (**a**) Profile after using time-domain SVD; (**b**) eliminated profile.

**Figure 12:**
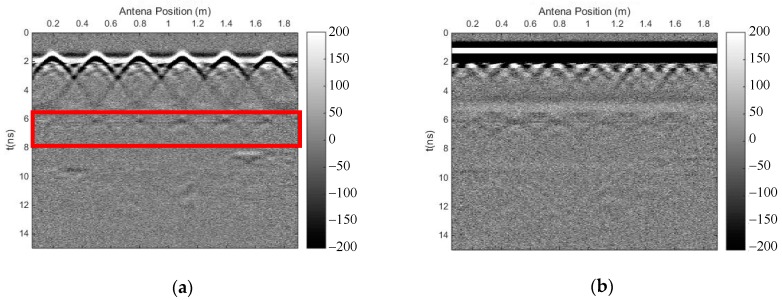
The results of the SVD method based on the Hankel matrix in the local frequency domain. (**a**) Profile after using the proposed method; (**b**) eliminated profile.

We carried out both methods to eliminate the strong direct wave and the reflected signal of the surface, and to suppress random noise. Figure 11 shows the profile after using the time-domain SVD method, and the eliminated profile. It can be seen that there is a small horizontal fake signal on top of each reflected signal of the rebar, which influenced the recognition of the travel time; moreover, this method introduced other horizontal fake signals. Figure 12 shows the profile after using the SVD method based on the Hankel matrix in the local frequency domain, and the eliminated profile. This method did not introduce horizontal fake signals over each reflected signal of the rebar, but the effective signal was also damaged. Similarly, in the regions where fake signals were introduced on top of each reflected signal of the rebar in Figure 11a, the power of the signals including the fake signals was calculated for comparison with the same regions in Figure 12a. The ratio was 2.9, indicating that the new proposal introduced much fewer fake signals than the time-domain SVD method. Meanwhile, the method could also weaken some multiple waves of the rebar, as seen in the red frame in Figure 12a. In the region with multiple waves of the rebar in Figure 11a, the power of the signals was calculated for comparison with the same region in Figure 12a. The ratio was 1.02, indicating that the newly proposed method could also weaken some multiple waves.

### 3.2. Real Field Data

#### 3.2.1. Underwater Archaeological Investigation

**Figure 13:**
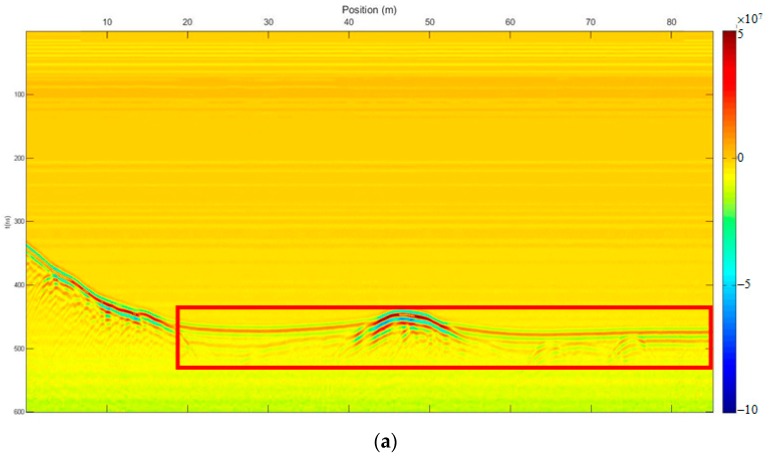

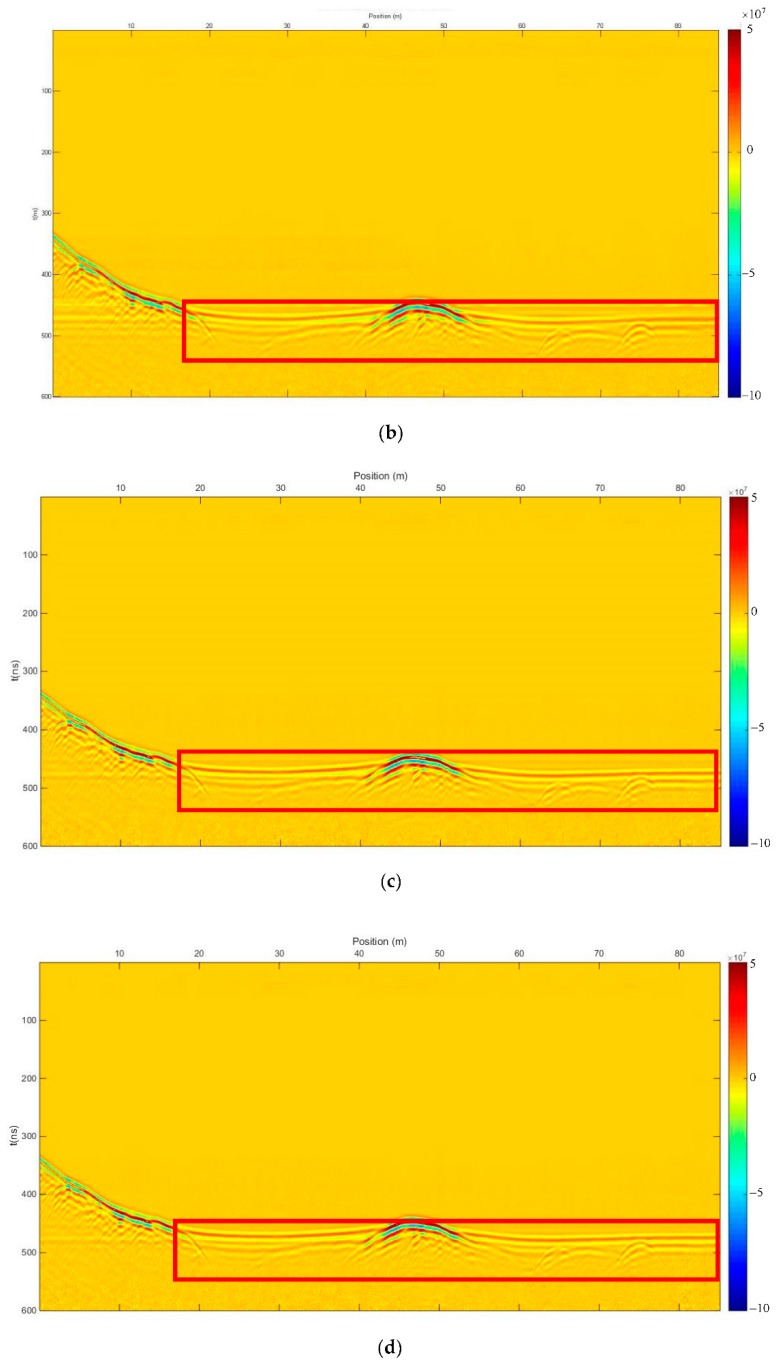
The results of the field data from an underwater archaeological investigation. (**a**) Measured profile; (**b**) profile after using the SVD method based on the Hankel matrix in the local frequency domain; (**c**) profile after using the time-domain SVD method; (**d**) profile after mean-trace removing.

GPR is widely used for archaeological investigation on land because it transmits non-destructive electromagnetic waves into the ground without interference on surrounding cultural relics, and it sends images of the targets in shallow subsurfaces with high lateral and vertical resolution. According to the research of Qin and Zhao et al. [5], GPR can also be used in underwater archaeology, although some may think it infeasible because electromagnetic waves would be quickly absorbed by water. Actually, the depth of penetration and the resolution of the signal might be even better than on land if the electrical conductivity of the water is not too high. They carried out an experiment using antennas of different frequencies in a particular area of the Shanglinhu Lake in Ningbo, well known as the origin of ceramics, to detect ancient kiln remnants and other cultural relics flooded and eventually covered by lacustrine sediments. Figure 13a shows a profile of the GPR field data measured at the lake, with several horizontal signals, perhaps representing multiple waves. These horizontal signals may influence the recognition of some effective signals of the cultural relics. We use the SVD method based on the Hankel matrix in the local frequency domain to eliminate these signals and the background noise, resulting in the profile shown in Figure 13b. It is obvious that the reflected signals of some objects at the bottom of the lake in the red frame are much clearer, and the horizontal signals are almost eliminated, allowing easier recognition of the lakebed and more targets underwater. Figure 13c shows the result after using the time-domain SVD method, and Figure 13d shows the result after mean-trace removing, both of which also eliminated clutter and background noise. However, the reflected signals of some objects at the bottom of the lake in the red frame in Figure 13c,d are not as clear as that in Figure 13b, due to stronger noise. Therefore, the proposed method performed better than the time-domain SVD method and mean-trace removing when eliminating clutter and noise in this case.

#### 3.2.2. Airport Runway Foundation Evaluation

**Figure 14:**
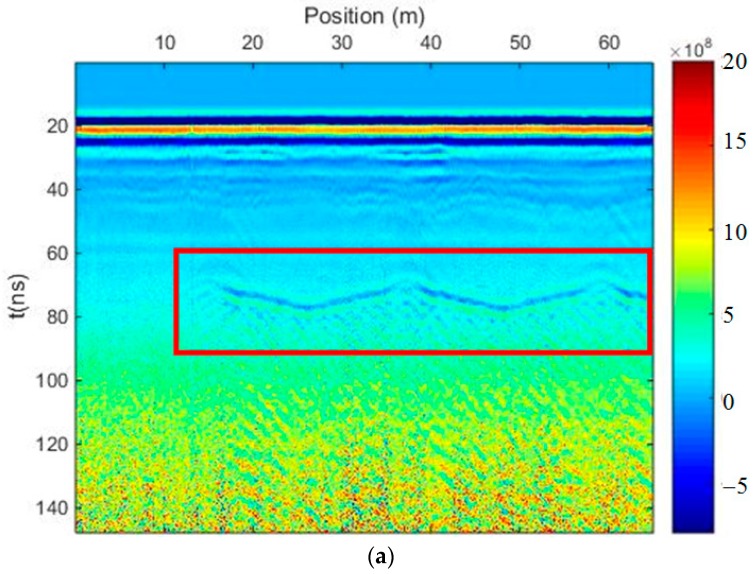

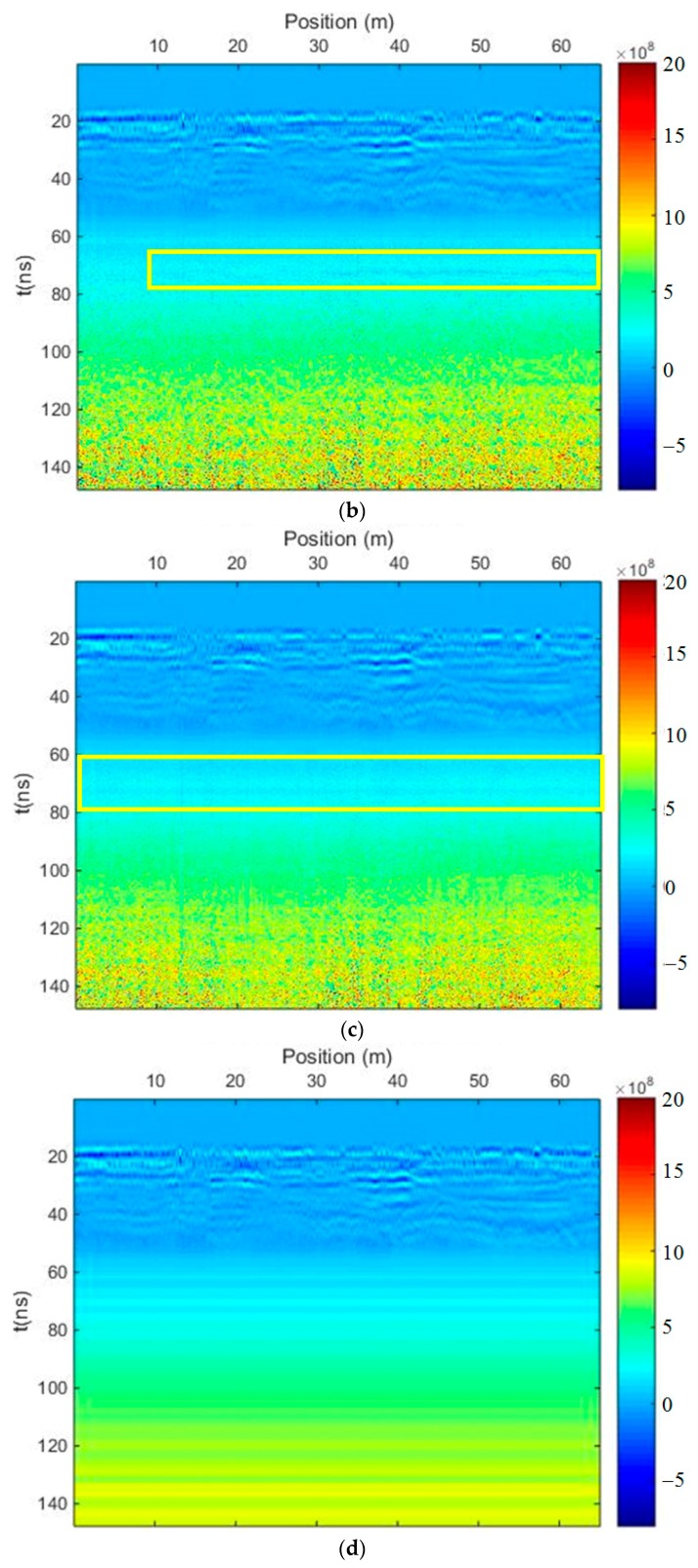
The results of the field data from the exploration of a runway in an airport. (**a**) Measured profile; (**b**) profile after using the SVD method based on the Hankel matrix in the local frequency domain; (**c**) profile after using the time-domain SVD method; (**d**) profile after mean-trace removing.

Figure 14a shows a profile of the GPR field data measured on a runway in an airport, with a strong direct wave and a reflected wave of the ground surface covering the signals of shallow targets underground. Furthermore, due to the reflection of hangar ceilings, there is a lot of regular clutter that is obvious in the middle of the profile, which seriously influences the recognition of other effective signals. There is a layered soil medium below the runway; thus, the clutter may cover weak signals reflected from the deep interface. Based on SVD research, and considering that the clutter from the hangar ceiling may correspond to a different singular value from the effective signals of the horizontal interfaces underground, yet may correspond to the same singular value as some effective reflected signals in the shallow layer, we divided the profile into an upper and lower region. We can determine the time window of the clutter from the hangar ceiling in the profile; therefore, the interface of the upper and lower regions was set at *t* = 55 ns. We needed to eliminate the direct wave and the reflected wave of the ground surface with strong auto-correlation in the upper region of the profile using the proposed method. In the lower region, we first extracted the clutter from the hangar ceiling using the proposed method and selected the appropriate singular values, before subtracting the clutter data from the lower region. Finally, we merged the upper and lower regions to get the profile in Figure 14b after filtering. Many target signals can be seen in the shallow layer, and a reflected signal is shown in the yellow frame, which may indicate an interface underground. Figure 14c shows the result of the time-domain SVD method using the same process, with a complete horizontal phase axis in the yellow frame. However, there is no clear signal in the region encompassing 72 ns (*y*-axis) and 0–10 m (*x*-axis) of the profile in Figure 14a; thus, the time-domain SVD method introduced a fake signal into the profile when suppressing the clutter. Figure 14d shows the result of mean-trace removing with the same process. There was no big difference among the three results in upper region of the profile, but there were many fake signals introduced into the lower region of Figure 14d because we extracted the background signal to delete the clutter from the hangar ceiling.

## 4. Discussion and Conclusions

Compared with the conventional time-domain SVD method and mean-trace removing, the SVD method based on the Hankel matrix in the local frequency domain of GPR data could weaken the horizontal fake signals introduced by eliminating the direct wave, and it could improve suppression of random noise around non-horizontal phase reflection events. The re-constructed GPR profile using the new SVD method could, thus, provide more accurate results. However, the comparison of the simple-layer model experiment also revealed that the newly proposed SVD method is not effective when removing noise, and results in little damage to the effective signals. When the energy of the effective signal is not very weak, this method is acceptable to a certain degree. In some complex cases, it may be an effective method to divide the GPR data into several regions to suppress specific noise, such as interference clutter from ground objects. The elimination of clutter (ground objects) in the GPR detection of an airport runway was successfully applied.

The SVD method based on the Hankel matrix in the local frequency domain needs to be optimized in future work to achieve better filtering when eliminating clutter, to improve the suppression of random noise, and to weaken the damage to effective signals.
